# Sequestration of Alkyltin(IV) Compounds in Aqueous Solution: Formation, Stability, and Empirical Relationships for the Binding of Dimethyltin(IV) Cation by N- and O-Donor Ligands

**DOI:** 10.1155/2009/219818

**Published:** 2009-07-05

**Authors:** Agatino Casale, Concetta De Stefano, Giuseppe Manfredi, Demetrio Milea, Silvio Sammartano

**Affiliations:** Dipartimento di Chimica Inorganica, Chimica Analitica e Chimica Fisica, Università di Messina, Salita Sperone, 31, 98166 Messina, Italy

## Abstract

The sequestering ability of polyamines and aminoacids of biological and environmental relevance (namely, ethylenediamine, putrescine, spermine, a polyallylamine, a branched polyethyleneimine, aspartate, glycinate, lysinate) toward dimethyltin(IV) cation was evaluated. The stability of various dimethyltin(IV) / ligand species was determined in NaCl_aq_ at *t* = 25°C and at different ionic strengths (0.1 ≤ *I*/mol L^−1^ ≤ 1.0), and the dependence of stability constants on this parameter was modeled by an Extended Debye-Hückel equation and by Specific ion Interaction Theory (SIT) approach. At *I* = 0.1 mol L^−1^, for the ML species we have log *K* = 10.8, 14.2, 12.0, 14.7, 11.9, 7.7, 13.7, and 8.0 for ethylenediamine, putrescine, polyallylamine, spermine, polyethyleneimine, glycinate, lysinate, and aspartate, respectively. The sequestering ability toward dimethyltin(IV) cation was defined by calculating the parameter pL_50_ (the total ligand concentration, as −log C_L_, able to bind 50% of metal cation), able to give an objective representation of this ability. Equations were formulated to model the dependence of pL_50_ on different variables, such as ionic strength and pH, and other empirical predictive relationships were also found.

## 1. Introduction

The knowledge of the behavior of organotin(IV) cations in the environment is of great concern for many scientists in several different research fields. The importance of these compounds, from different points of view, was already extensively discussed (e.g., [1–13]). Their environmental and biological activity is mainly related to their chemicophysical behavior in aqueous solution. In fact, their aqueous chemistry is dominated by the formation of various hydrolytic species, even if they also tend to interact with several organic and inorganic ligands, forming a wide number of complex species of different stability. This is particularly relevant in the study of organotin(IV) speciation in natural and waste waters and biological fluids, where other metals and various organic (carboxylic and aminic in particular) and inorganic ligands could be simultaneously present in different concentrations (see, e.g., in [8, 14–18]). In fact, it is well known that organotin(IV) compounds show different biological and environmental activity depending on their speciation: the formation of different species plays an important role in organotin(IV) toxicity and exposure to living organisms and influences their availability, their accumulation, biomodification, and their transport inside the organisms and within and between various environmental compartments [8, 9, 11, 15, 18, 19]. 

Owing to the objective impossibility of defining the speciation and the sequestration of organotin(IV) compounds in all the different systems where they could be present, since some years we undertook a study on their interactions with various ligand classes, in order to derive general information and empirical relationships to be used for the prediction of both the chemicophysical behavior and the sequestering ability of these ligands toward organotin(IV) cations (e.g., [16, 18–21]. For example, in some of our previous papers we derived some empirical relationships for the modeling of the stability of diethyltin(IV) complexes with O- and N-donor ligands [16], whilst in others we modeled that of mono-, di-, and trialkyltin(IV) complexes with various carboxylic ligands as a function of simple ligand and metal structural parameters (e.g., the charge of the alkyltin(IV) cation, the number and nature of binding sites, etc.) [19]. 

At the same time, the choice of N-donor ligands (aminoacids and polyamines) was supported by the fact that, despite their importance and their massive presence in natural waters and biological fluids, reported thermodynamic data (stability constants, formation enthalpies, and entropies…) on their interactions with alkyltin(IV) cations are limited (e.g., [7, 16, 22–32]) with respect to contributions on alkyltin interactions with other ligands such as, carboxylates (carefully analyzed, e.g., in [15]). Furthermore, an accurate analysis of some of those papers evidences that alkyltin(IV) cations preferably bind to ligands via nitrogen groups rather than via oxygen. For example, in the case of lysine and ornithine, which may coordinate as bidentate ligands either by (N, N) or (N, O) donor sets, there is evidence that they bind to dimethyltin(IV) by the former (N, N) donor set [24]. 

Since natural waters and biological fluids cover a very wide range of ionic strengths (from *I* ~ 0.01 mol L^−1^ for spring waters to *I* > 6 mol L^−1^ for hyper-saline waters), stability constants of various dimethyltin(IV) species were determined in NaCl_aq_ at *t* = 25°C and at different ionic strengths, and their dependence on this parameter was modeled by an Extended Debye-Hückel equation and by Specific ion Interaction Theory (SIT) approach [33–35]. Finally, several values of pL_50_ (the total ligand concentration, as −log *C*_*L*_, able to bind 50% of metal cation), an empirical parameter used to give an objective representation of the sequestering ability of a ligand [36–38], were calculated for the sequestration of various ligands toward dimethyltin(IV) cation. Equations were formulated to model the dependence of pL_50_ on different variables (e.g., ionic strength and pH), and other empirical predictive relationships were also found between the stability of complexes and the kind and number of functional groups of the ligand(s) involved in the formation equilibria. 

In the present paper, we extended this study to the evaluation of the sequestering ability of polyamines and aminoacids of biological and environmental relevance toward dimethyltin(IV) cation. We opted for the dimethyltin(IV) cation since it is one of the main representatives of diorganotin(IV) compounds. The actual, renewed interest in the chemistry of diorganotin(IV) derivatives is due to the fact that, despite they are less toxic than triorganotin(IV) cations, more recent researches (e.g., [3, 39]) suggest them to possess anticarcinogenic activity, in contrast with the suspected carcinogenicity of other organotin(IV) compounds (triderivatives first) [7, 11]. 

## 2. Experimental Section

### 2.1. Chemicals

Dimethyltin(IV) [(CH_3_)_2_Sn^2+^, *dmt*] dichloride (Alfa-Aesar) was used without further purification, and its purity was checked potentiometrically by alkalimetric titrations, resulting always ≥99%.1, 2-diaminoethane (ethylenediamine, *en*), 1,4-diaminobutane (putrescine, *ptr*), N, N′-bis(3-aminopropyl)-1,4-butanediamine (spermine*, sper*), polyallylamine (MW ~ 15 kDa, *paam*), and branched polyethyleneimine (MW ~ 750 kDa, *pei*) were used in their hydrochloride forms (di-, di-, tetra-, poly-, and poly- for *en*, *ptr*, *sper*, *paam*, and *pei*, resp.). Aspartate (*asp* ^2−^) and glycinate (*gly* ^−^) were used as L-aspartic acid and glycine, respectively; lysinate (*lys* ^−^) was used as L-lysine hydrochloride. All ligands were of analytical grade and were purchased from Sigma-Aldrich (and its various brands). They were used without further purification, and their purity was checked potentiometrically by alkalimetric titrations, resulting always ≥99%. Hydrochloric acid and sodium hydroxide solutions were prepared by diluting concentrated ampoules (Riedel-deHaën) and were standardized against sodium carbonate and potassium hydrogen phthalate, respectively. NaOH solutions were preserved from atmospheric CO_2_ by means of soda lime traps. NaCl aqueous solutions were prepared by weighing pure salt (Fluka) dried in an oven at 110°C. All solutions were prepared with analytical grade water (R = 18 M cm^−1^Ω) using grade A glassware.

### 2.2. Apparatus and Procedure

Potentiometric measurements were carried out (at *t* = 25.0 ± 0.1°C) using an apparatus consisting of a Model 713 Metrohm potentiometer, equipped with a combination glass electrode (Ross type 8102, from Thermo/Orion), or a half cell glass electrode (Ross type 8101, from Thermo/Orion) and a double junction reference electrode (type 900200, from Thermo/Orion), and a Model 765 Metrohm motorized burette. Estimated precision was ±0.15 mV and ±0.003 mL for e.m.f. and titrant volume readings, respectively. The apparatus was connected to a PC, and automatic titrations were performed using a suitable computer program to control titrant delivery and data acquisition and to check for e.m.f. stability. Some measurements were also carried out using a Metrohm model 809 Titrando apparatus controlled by Metrohm TiAMO 1.0 software for the automatic data acquisition. Potentiometric titrations were carried out in thermostatted cells under magnetic stirring and bubbling purified presaturated N_2 _ through the solution in order to exclude O_2_ and CO_2_ inside. The titrand solution consisted of different amounts of dimethyltin(IV) dichloride (0.8–3 mmol L^−1^), ligand (0.8–5 mmol L^−1^), a slight excess of hydrochloric acid (0.8–5 mmol L^−1^), and the background salt in order to obtain pre–established ionic strength values (0.1 ≤ *I* mol L^−1^ ≤ 1.0; 0.1 and 0.5 mol L^−1^ for *gly* and *lys*). The most of measurements were performed considering an M : L = 1 : 1 metal to ligand ratio, except for some where M : L = 1 : 2. Potentiometric measurements were carried out by titrating 25 mL of the titrand solution with standard NaOH solutions up to pH ~ 8.5–9. However, since the formation of sparingly soluble species was never observed in the experimental conditions adopted, some titrations were performed up to pH ~ 10.5–11. For each experiment, independent titrations of strong acid solution with standard base were carried out under the same medium and ionic strength conditions as the systems to be investigated, with the aim of determining electrode potential (*E* ^0^) and the acidic junction potential (*E*_*j*_ = *j*_*a*_[H^+^]). In this way, the pH scale used was the total scale, pH ≡ −log [H^+^], where [H^+^] is the free proton concentration (not activity). The reliability of the calibration in the alkaline range was checked by calculating p*K*_w_ values. For each titration, 80–100 data points were collected, and the equilibrium state during titrations was checked by adopting some usual precautions. These included checking the time required to reach equilibrium and performing back titrations.

### 2.3. Calculations

The nonlinear least squares computer program ESAB2M [40] was used for the refinement of all the parameters of the acid-base titration (*E* ^0^, *K*_w_, liquid junction potential coefficient, *j*_*a*_, analytical concentration of reagents). The BSTAC [41] and STACO [42] computer programs were used in the calculation of complex formation constants. Both programs can deal with measurements at different ionic strengths. The ES4ECI [41] program was used to draw speciation and sequestration diagrams and to calculate species formation percentages. The LIANA [43] program was used to fit different equations.

Protonation, hydrolysis, and complex formation constants are given according to the equilibria (M = *dmt* and L = fully deprotonated ligand):

(1) $$p\;{\text{M}}^{2+}\;+\;q\;{\text{L}}^{\text{z}}\;+\;\text{r}\;{\text{H}}^{+}={\text{M}}_{p}{\text{L}}_{q}{{\text{H}}_{\text{r}}}^{\left(2p+q\text{z}+r\right)},\;\beta_{pqr},$$

(2) $${\text{M}}^{2+}\;+\;{\text{H}}_{r}{\text{L}}^{\left(z+\text{r}\right)}=\;\text{ML}{{\text{H}}_{\text{r}}}^{\left(2+z+\text{r}\right)},\;K_{11r},$$

(3) $$\text{M}{\left(\text{OH}\right)}^{+}+{\text{L}}^{z}=\text{ML}{\left(\text{OH}\right)}^{\left(z+1\right)},\;K_{11-1},$$

(4) $${\text{L}}^{z}\;+\;\text{ML}{{\text{H}}_{\text{r}}}^{\left[2+z+r\right]}=\;{\text{ML}}_{2}{{\text{H}}_{r}}^{\left(2+2z+r\right)},\;\;K_{12r}.$$

Dependence on ionic strength of stability constants of various species, expressed in the molar (mol L^−1^) concentration scale, was taken into account by a Debye-Hückel type equation:

(5) $$\log\;\;K_{pqr}={\log\;}^{\text{T}}K_{pqr}+\text{DH}+C\;I,$$

where *C* is an empirical parameter, and DH is the Debye-Hückel term that, at *t* = 25°C, with *A* = 0.51 and *åB* = 1.5, is given by

(6) $$\text{DH}=\frac{{-z}^{*}\;0.51\;I^{1/2}}{\left(1\;+\;1.5\;I^{1/2}\right)},$$

with

(7) $$z^{*}=\Sigma \;{\left(\text{charges}\right)}_{\text{reactants}}^{2}-\Sigma {\left(\text{charges}\right)}_{\text{products}}^{2}.$$

The dependence on medium and on ionic strength of equilibrium thermodynamic parameters has been also taken into account by the Specificion Interaction Theory (SIT) model [33–35]. By using appropriate density values [44], molar to molal [*m*, mol kg^−1^ (H_2_O)] scale conversions of *I* and *K*_pqr_ were performed. When these are expressed in the molal concentration scale, (5) becomes the classical SIT equation [33–35], where *C* is replaced by ∆ε:

(8) $$∆\varepsilon =\;{\Sigma \varepsilon }_{\text{i}}\left(i,\;j\right)$$

The *ε*(*i*, *j*) parameter is the SIT interaction coefficient of the *i*th species (involved in the equilibrium represented by the formation constant *K*_*pqr*_) with the *j*th component (of opposite charge). ∆ε parameters as well as single interaction coefficients *ε*(*i*, *j*) were determined too.

## 3. Results and Discussion

### 3.1. Dimethyltin(IV) Hydrolysis and Ligand Protonation

Prior to any study of the binding ability of different ligands toward dimethyltin(IV) cation, an accurate knowledge of the acid-base behavior of both the ligands and *dmt* is necessary. Protonation constants of polyamines and aminoacids, as well as dimethyltin(IV) hydrolysis constants, were already determined in several experimental conditions, together with the parameters for the modeling of their dependence on medium, ionic strength, and temperature [45–57]. As an example, in Table 1 some of these values are reported, in NaCl_aq_ at *I* = 0.1 mol L^−1^ and *t* = 25°C. In the analysis of this table, it is important to make a brief comment on the protonation constants of *paam* and *pei*. Previous studies [58] demonstrated that, in addition to the classical models used to describe the acid-base behavior of polyelectrolytes (e.g., Högfeldt [59]), these two polyamines can be considered as a low molecular weight diamine (*paam*) and a tetraamine (*pei*). In this way, all calculations and experiments are designed and performed by taking into account the simple dimeric and tetrameric units, respectively. This new model not only maintains the same degree of accuracy of the “classical” ones but also has the evident advantage of facilitating calculations (allowing, e.g., the use of the same computer programs). Furthermore, comparisons between these two polyamines and the used low molecular weight ligands are more immediate, from the point of view of both their acid-base behavior and their binding ability toward dimethyltin(IV) or any other compound.

### 3.2. Formation and Stability of Dimethyltin(IV)/Amine Species

Calculations performed on potentiometric data of *dmt*/amine systems gave evidence of the formation of the ML and MLH species for all considered amines. In all investigated systems, further ML_*q*_H_*r*_ species were formed, with different values of *q *(*q* = 1 or 2) and *r *(*r* = 2 or 3), depending on the ligand. Values of stability constants determined are reported in Table 2 for all ML_*q*_H_*r*_^(2+*r*)^ species in each system, at different ionic strengths. This table shows that the ML_2_^2+^ species is only formed by the two low molecular weight diamines (i.e., *en* and *ptr*), whilst the polyallylamine (another diamine according to the model) forms the ML_2_H^3+^. On the contrary, as expected, spermine and polyethyleneimine (the two tetraamines) form two further protonated species, namely, MLH_2_^4+^ and MLH_3_^5+^. Among the investigated diamines, putrescine complexes are much stronger than the corresponding ones of ethylenediamine, whilst *paam* shows an intermediate behavior. Analogously, species formed by spermine are more stable than those by polyethyleneimine. Globally, the stability of the simple ML species formed by *dmt* with all investigated amines follows the trend

*sper* > *ptr* > *paam* ~ *pei* > *en*, 

whilst a slight different order is observed for the other common species, that is, MLH:

*sper* > *pei* > *ptr* ~ *paam* > *en*.

From the analysis of Table 2, another interesting aspect is worthy of mention. Among the two investigated low molecular weight diamines (i.e., *en* and *ptr*), the stability of ML species is evidently higher for *ptr* than for *en*. At first sight, this behavior appears puzzling, considering that ethylenediamine may form with dimethyltin(IV) cation a “five membered” chelate ring, which should be more stable than the analogue “seven membered” ring formed by putrescine. This fact may be interpreted considering that with quite “large” cations, such as organotin cations, ligands with longer alkyl chains (e.g., *ptr* instead of *en*) usually form stronger ML species than shorter ligands. With these very large cations, chelation by small ligands is disadvantaged for steric factors, so that these ligands tend to act as monodentate, with a very small contribution of the second N donor group. We also had the same evidence for the interactions of *en* and *ptr* with dioxouranium(VI) cation (unpublished work from this laboratory). For similar reasons, the analogies in the stability of the (*dmt*)(*ptr*) and (*dmt*)(*sper*) species should be an indication that not all the four spermine amino groups are involved in the coordination to dimethyltin(IV). However, further spectroscopic studies were planned to verify these hypothesis and will be the subject of another contribution.

### 3.3. Formation and Stability of Dimethyltin(IV)/Aminoacid Species

In order to give a more detailed picture of the binding ability of O- and N-donor ligands toward dimethyltin(IV), the speciation of this cation in the presence of three different aminoacids (i.e., glycine, lysine, and aspartic acid) was also investigated. As can be easily noted, in addition to the simplest aminoacid (i.e., glycine), one containing an extra amino group (i.e., lysine) and one with another carboxylic group (i.e., aspartic acid) were selected. Experimental data analysis revealed that all the three ligands form with dimethyltin(IV) cation three main species, namely, ML^(2+*z*)^, MLH^(3+*z*)^, and the hydroxo-species ML(OH)^(1+*z*)^. In addition to these species, only lysine forms another protonated species, the MLH_2_^3 + ^. Corresponding stability constants are reported in Table 3, at the investigated ionic strengths. As can be observed from the analysis of this table, for all the common species, the order of their stability is

(9) lys≫asp~gly.

### 3.4. Influence of Ligand Complexes on Dimethyltin(IV) Speciation

**Figure 1:**
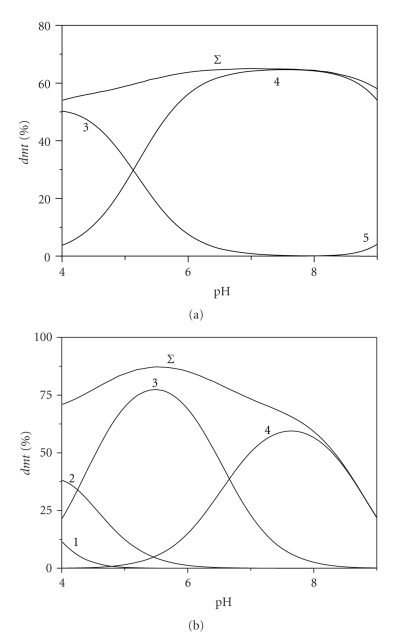
Distribution diagrams of dimethyltin(IV)/amine species versus pH in NaCl_aq_ at *t* = 25°C. Diagrams: (a) putrescine; (b) polyethyleneimine (considered as a simple diamine). Species: (1) MLH_3_; (2) MLH_2_; (3) MLH; (4) ML; (5) ML_2_. Charges omitted for simplicity. Experimental conditions: * C*_*dmt*_ = 0.003 mol L^−1^, *C*_*L*_ = 0.005 mol L^−1^; *I* = 0.1 mol L^−1^.

**Figure 2:**
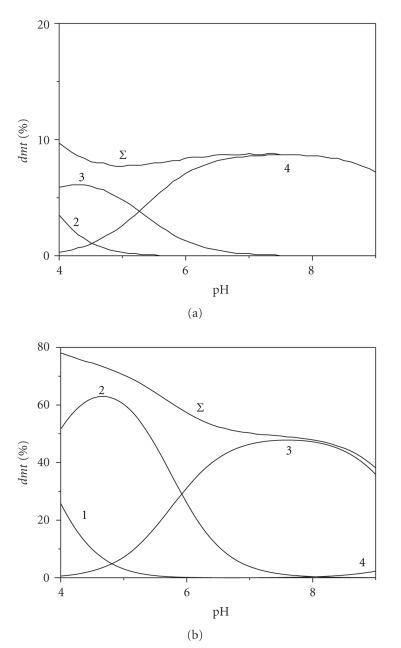
Distribution diagrams of dimethyltin(IV)/aminoacid species versus pH in NaCl_aq_ at *t* = 25°C. Diagrams: (a) glycinate; (b) *L*-lysinate. Species: (1) MLH_2_; (2) MLH; (3) ML; (4) MLOH. Charges omitted for simplicity. Experimental conditions: * C*_*dmt*_ = 0.003 mol L^−1^, *C*_*L*_ = 0.005 mol L^−1^; *I* = 0.1 mol L^−1^.

The importance of dimethyltin(IV) complexes with the investigated O- and N-donor ligands on its speciation can be appreciated looking at Figures 1  and 2  where, for example, the percentages of species formed by this cation with two amines (*ptr* and *pei*) and two aminoacids (*gly* and *lys*) are reported in NaCl_aq_ at *I* = 0.1 mol L^−1^ and *t* = 25°C. As can be noted from these Figures, *dmt */ligand species are formed in the whole investigated pH range, with percentages ranging from ~10% to ~80%. In particular, the highest values are observed for polyethyleneimine species, whilst the lowest value regard complexes formed by glycinate and aspartate. This is a first indication that dimethyltin(IV) cation forms stronger species with N-donor groups than with O-donor. In fact, among the three investigated aminoacids, formation percentages of lysinate species (contain an extra amino-group) are three-four times those reached by glycinate (and aspartate). Worth mentioning is also that, increasing pH, the percentage of dimethyltin complexed by polyamines first increases (more or less sharply, depending on the ligand) and, after a maximum, it decreases. This is due to the fact that, at low pH, investigated polyamines are partially or totally protonated, and their binding ability is significantly reduced. Nevertheless, in the basic pH range, the formation of hydrolytic species (mainly the neutral *dmt*(OH)_2_) is so strong that it inhibits complexation. This trend is less marked for aminoacids, where the carboxylic group is already deprotonated at low pHs.

### 3.5. Dependence on Ionic Strength of Dimethyltin(IV) Species

**Figure 3:**
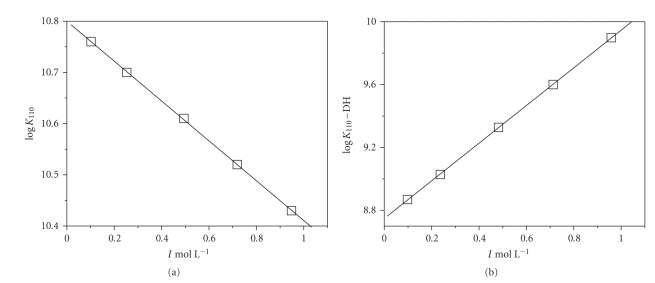
Dependence of stability constants of (*dmt*)(*en*)^2+^ (as log *K*_110_) and (*dmt*)(*asp*) (as log *K*_110_ − DH) species on ionic strength (in mol L^−1^), in NaCl_aq_ at *t* = 25°C.

Stability constants of dimethyltin(IV) complexes reported in Tables 2  and 3  proved fairly dependent on ionic strength, as shown in Figure 3 where, for example, log *K*_110_ values for en and asp are plotted as a function of *I*, in mol L^−1^ (stability constants referred to reactions with z* ≠ 0, such as log *K*_110_ of (*dmt*)(*asp*), are usually plotted as log *K* − *DH*). The lines in the same figure represent the dependence on ionic strength expressed by (5), where *I* = 0.1 mol L^−1^ is taken as reference ionic strength. Refined parameters of this equation are reported in Tables 4  and 5, for species formed by amines and aminoacids, respectively. Of course, parameters related to the dependence on ionic strength of glycinate and lysinate species, based on two ionic strengths only, have no mathematical meaning. Nevertheless, if simultaneously analyzed with those of other systems, these parameters can evenly give a general picture of the dependence on ionic strength of these complexes. In the same tables, corresponding refined ∆ε parameter is reported for the fitting of stability constants converted in the molal scale. Since differences in the refined log *K*_pqr_ at *I* = 0.1 mol L^−1^ and *I* = 0.1 mol kg^−1^(H_2_O) resulted lower than the error associated to this parameter, only a common value was reported in Tables 4  and 5, valid for both molar and molal datasets. Formation constants and ionic strength values reported in Tables 2  and 3  were converted into the molal (*m*, mol kg^−1^(H_2_O)) concentration scale (data shown in Tables 6  and 7, for *dmt*/amine and *dmt*/aminoacid species, resp.) with the aim of modeling the dependence of stability constants of dimethyltin(IV)-ligand species on ionic strength also by the SIT equations, in order to determine SIT interaction coefficients for these species. From the simultaneous analysis of all datasets by LIANA program, classical SIT interaction coefficients of species involved in protonation, hydrolysis, and complex formation equilibria were equally derived and are shown in Table 8 (except for those regarding *gly* and *lys*). Water activity and interaction coefficients among proton and chloride ions were taken from literature [60]. Calculations of interaction coefficients reported in Table 8 were only possible fixing some values (otherwise the system is mathematically undetermined): preliminary analysis evidenced that coefficients related to the fully deprotonated, neutral, polyamines were close to “0”, and, for this reason, in successive calculations these values were considered as fixed, and this choice is coherent with the original SIT theory, where only interactions between ions of opposite sign are taken into account. On the other hand, it is possible to use “nonzero” coefficients for the interactions of neutral species with the ionic medium, as suggested by several authors (see, e.g., [35] and references therein). Hence, the SIT theory has the potential to describe the activity coefficient and related properties of neutral species [35]. This is the case, for example, of LH_2_ and ML species of aspartate, reported in Table 8.

### 3.6. Sequestering Ability of Various Ligands toward Dimethyltin(IV) Cation

We already stressed that the sequestration of metal and organometal cations in natural fluids and waste waters plays a very important role, both negative and positive, in many fields. Few examples of positive effects include the use of chelating agents in chelotherapy; the interaction of some ligands with calcium to solubilize urinary stones; the sequestration of toxic metals in waste waters; the sequestration of some essential metals to favor their uptake by plants. Cases of negative effects are represented by the removal of heavy metals from sediments, with consequent mobilization; by the sequestration of essential metals in chelotherapy; by the formation of metal-ligand species more toxic than the metal itself. All these effects can be correctly taken into account by equilibrium speciation analysis, using suitable approaches, calculation methods, and efficient models.

Different level problems are involved in sequestration studies. In the first level it must be taken into account (a) the variety of composition and temperature of different fluids and (b) the need to find reliable parameters to quantitatively express the efficiency of different sequestering agents. The first problem requires (1) the formulation/use of models for the dependence on ionic strength/ medium/temperature of equilibrium parameters for the formation of different species in the considered system; (2) the use of appropriate and correct datasets; (3) when some parameters are not available, to build robust means for their prediction. The second problem is related to the network of interactions that occur in a multicomponent system and in particular (1) to the different complexing abilities of different ligand classes in different conditions and (2) to the competition of the proton and/or OH^−^with metals and ligands involved in the sequestration process. By analyzing the stability of some classes of complexes, remarkable differences may be observed. Nevertheless, a significant difference in the stability of two complexes does not always imply significantly different sequestration power in a real system, owing to the interactions between the metal with other ligands and the ligand with other metals. Also the medium effect plays an important role, for example, by increasing ionic strength, proton-amine and metal-amine formation constants usually show an opposite trend with respect to analogous carboxylate species [61, 62]. In other words, comparing infinite dilution or high constant ionic strength formation constants may lead to quite different results. Even considering a very simple one-metal/one-ligand system, the competition of H^+^ with the ligand and OH^−^ with the metal must be taken into account. The comparison of the stability of a metal chelate with a very basic ligand to that of another metal chelate with a moderate basic ligand does not give a measure of the sequestering power of the two ligands. 

For this reason, recently a simple parameter was proposed to have a measure of the sequestering ability of a ligand toward different metal ions or different ligands toward a metal ion [36–38]. This is an empirical parameter that, once the conditions (pH, ionic strength, supporting electrolyte, temperature) are fixed, can give an objective representation of the binding ability. A detailed description of the method is given, for example, in [36–38]. Briefly, pL_50_ represents the total ligand concentration (as antilogarithm) necessary to bind 50% of cation in solution (as trace) and is obtained by the Boltzman type equation:

(10) $$y=\frac{A_{1}-A_{2}}{1+e^{(\text{pL}-\text{p}{\text{L}}_{50})/\text{S}}}+{\text{A}}_{2},$$

where *y* represents the total percentage of notcomplexed metal (*dmt* in our case), *A*_1_ = 0 and *A*_2_ = 100, and *S* is the curve slope at 50% complexation. In other words, the higher the pL_50_ is, the stronger the binding ability of the ligand toward dimethyltin(IV) is. 

**Figure 4:**
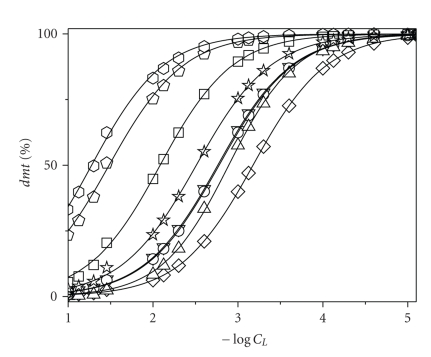
Dimethyltin(IV) sequestration diagrams in presence of various ligands. Percentage of noncomplexed *dmt* as a function of total ligand concentration (as −log *C*_*L*_) at *I* = 0.1 mol L^−1^ in NaCl_aq_ and pH = 6.50. Total concentration of *dmt*, *C*_*dmt*_ = 10^−9^ mol L^−1^. Symbols: 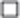: * en*; 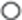: * ptr*; 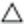: * paam*; 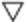:*sper*; 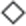:*pei*; 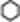:*gly*; 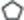:*asp*; 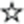:*lys*.

In Figure 4, some examples of *dmt* sequestration diagrams, used to derive pL_50_ values, are reported for all investigated ligands at *I* = 0.1 mol L^−1^ and pH = 6.5. Looking at this figure, it is immediately clear that the sequestering ability of investigated ligands toward dimethyltin cation follows the trend

(11) pei>paam>sper≅ptr>lys>en>asp>gly.

This order supports the statement that the binding abilities of various ligands cannot be only compared by the simple analysis of stability constants or just from structural considerations, such as the number of binding sites. In fact, for example, *pei* appeared to be a better sequestering agent toward *dmt* than *sper*, despite log *K*_110_ for (*dmt*)(*sper*)^2+^ species are higher than corresponding values for (*dmt)*(*pei*)^2+^. Analogously, a diamine like *ptr* shows in those conditions the same binding ability of a tetramine like *sper*. 

**Figure 5:**
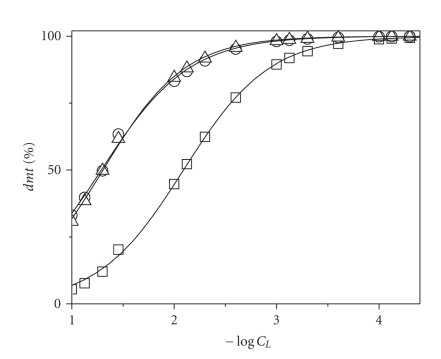
Dimethyltin(IV) sequestration diagrams in presence of ethylenediamine, glycinate, or malonate. Percentage of noncomplexed *dmt* as a function of total ligand concentration (as −log *C*_*L*_) at *I* = 0.1 mol L^−1^ in NaCl_aq_ and pH = 6.5. Total concentration of *dmt*, *C*_*dmt*_ = 10^−9^mol L^−1^. Symbols: 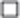: en; 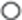: gly; 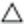: mal.

Curves in Figure 4 also better evidence what already observed from the analysis of both speciation diagrams and stability constants of various *dmt*/ligand systems, that is, that N-donor ligands better sequester *dmt* than O-donor. As a further confirmation, the *dmt* sequestration diagrams of ethylenediamine, glycinate, and malonate (*mal*, stability constants taken from [15]) are shown in Figure 5. These three ligands (similar because they represent difunctional compounds where the two groups are separated by just one “–CH_2_–”) are suitable for this kind of comparison because of their “systematic” differences: (i) malonate has two carboxylic groups in its structure, (ii) glycinate has one carboxylic and one aminogroup, and (iii) ethylenediamine has two aminogroups. As expected, the greatest sequestering ability toward *dmt* is shown by ethylenediamine.

### 3.7. Dependence of the Sequestering Ability on pH and Ionic Strength

As already pointed out, natural waters and biological fluids, as well as waste waters, show a great variability in their composition. Very important from an environmental and biological point of view are some fundamental parameters, such as temperature, ionic strength, and pH, whose variations also affect the sequestering power of various ligands. Previous studies on different systems [36, 37] showed that the greatest changes in pL_50_ (and, therefore, in the sequestration) very often occur when varying the last two parameters, whilst the effect of temperature is still present but is often less marked. In Tables 9  and 10, several pL_50_ are reported for all investigated ligands, at different pH and ionic strengths. 

**Figure 6:**
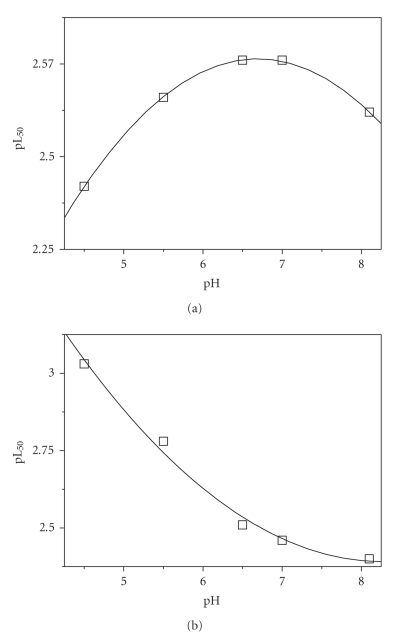
Dependence on pH of pL_50_ values for *dmt* sequestration by *sper* (a) and *lys* (b) at *I* = 0.1 mol L^−1^ in NaCl_aq _ at *t* = 25°C.

Despite the sequestering ability of a ligand and, therefore, pL_50_ is dependent on different conditions, this problem may be easily bypassed. In fact, one of the great advantages in the use of pL_50_ is that it may be often expressed as function of all the above cited variables by simple relationships. Also the sequestering ability of the investigated ligands toward dimethyltin(IV) cation may be easily modeled over a wide range of ionic strengths and pH. Some examples are represented by the dependence of pL_50_ for *sper* (12) and *lys* (13) on pH at *t* = 25°C and *I* = 0.1 mol L^−1^ (shown in Figure 6), given by

(12) $${\text{pL}}_{50}=\left(-0.45\pm 0.04\right)+\left(0.961\pm 0.01\right)\text{pH}+\left(-0.072\pm 0.001\right){\text{pH}}^{2},\;sper$$

(13) $${\text{pL}}_{50}=\left(5.5\pm 0.4\right)+\left(-0.8\pm 0.01\right)\text{pH}+\left(0.05\pm 0.01\right){\text{pH}}^{2},\;lys$$

**Figure 7:**
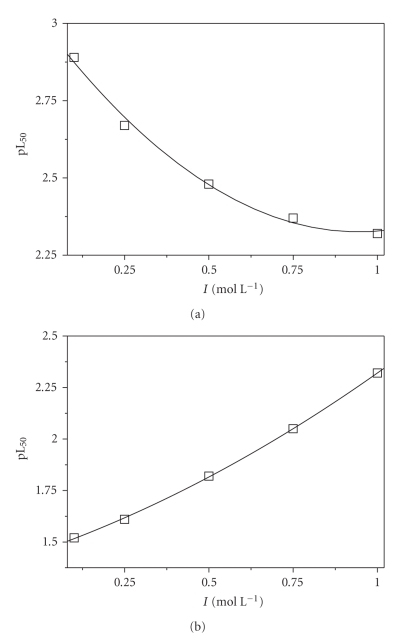
Dependence on ionic strength (in mol L^−1^) of pL_50_ values for *dmt* sequestration by *paam* (a) and *asp* (b) at pH = 6.5 and *t* = 25°C.

Analogously, pL_50_ for *paam* (14) and asp (15) at pH = 6.5 and *t* = 25°C may be expressed as a function of ionic strength (in mol L^−1^, Figure 7) by the relationships

(14) $${\text{pL}}_{50}=\left(3.01\pm 0.04\right)+\left(-1.5\pm 0.2\right)I+\left(-0.8\pm 0.1\right)I^{2},\;paam$$

(15) $${\text{pL}}_{50}=\left(1.456\pm 0.009\right)+\left(0.58\;\pm \;0.04\right)I+\left(0.29\pm 0.04\right)I^{2},\;asp$$

Other examples may be done, but those shown are also useful to remark again that various ligands may behave differently in terms of sequestration. For instance, looking at Figure 6 it is evident that the sequestering ability of spermine first increases with increasing pH and then decreases above pH ~ 7, where the formation of neutral hydrolytic *dmt*(OH)_2_ becomes significant, whilst pL_50_ for lysinate decreases regularly. In the same way, the sequestering ability of *paam* at pH = 6.5 regularly decreases increasing ionic strength, whilst that of *asp* shows an opposite trend (Figure 7).

### 3.8. Empirical Relationships for the Stability of Dimethyltin(IV)/Ligand Species

From the analysis of Tables 2  and 3, some systematic differences and regularities emerged in the stability of various *dmt*/amine and *dmt*/aminoacid species, suggesting the opportunity to find some useful relationships for the modeling of their behavior. This possibility is also supported by previous studies on the stability of various organotin/ligand systems, where several empirical relationships were found and used with predictive purposes (see, e.g., [16, 18, 19]). For example, the stability of some diethyltin(IV)/ligand species can be expressed as a function of the number of amino- and/or carboxylic groups in the ligand(s) involved in the formation reaction of these species [16]. Analogously, various thermodynamic parameters (log *K*, Δ*H*, *T*Δ*S*, Δ*G*) of several alkyltin(IV)/polycarboxylate species may be expressed as a function of the number of carboxylic groups in the ligand, the number of protons in the species, or the stability of other analogous metal/ligand complexes [19]. 

In this light, various attempts were made to find new useful correlations for the modeling of stability constants of dimethyltin(IV)/ligand species. Very interesting results were obtained when log *K*_11*r*_ of *dmt*/ligand species are expressed as function of both the ligand protonation constants and the number N-(n_N_) or O-donor (n_O_) groups available for complexation by the ligand (i.e., the unprotonated groups). In particular, for *dmt*/amine species at *t* = 25°C and infinite dilution, shown in the first part of Table 11, we have

(16) $$\log\;\;K_{11r}=\left(0.88\pm 0.07\right)\log\;\;K_{01(r+1)}+\left(1.02\pm 0.10\right){\text{n}}_{\text{N}},$$

whilst, in the same conditions, for some carboxylic ligands (data were taken from [15] and shown in the second part of Table 11) we have

(17) $$\log\;\;K_{11r}=\left(0.42\pm 0.02\right)\log\;\;K_{01(r+1)}+\left(1.30\;\pm \;0.05\right){\text{n}}_{\text{O}}.$$

From a rapid comparison of these two relationships, the marked difference in the stability of dimethyltin(IV) complexes with carboxylates and amines emerges, in great favor of the last ligands. For example, simple diamines and dicarboxylates (i.e., n_N_ = n_O_ = 2) generally have a mean value for the first protonation constant of log *K*_011_ ~ 10 and log *K*_011_ ~ 5, respectively, leading to a difference in the stability constant of the corresponding *dmt*/ligand species of ~5 log units. As expected, this difference sensibly decreases for protonated species, due to the presence of a positive charge in the protonated amine. On the basis of these results, however, the complexation behavior of these two ligand classes shows that dimethyltin(IV) cation is “border line” in the hard-soft scale. Similar conclusions were already reached also for the diethyltin(IV) cation (*det*) in a previous work [16], where the stability of various* det*–L–H species with N- and/or O-donor groups was calculated. From results obtained in the present paper it also appears that aminoacids show an intermediate behaviour between polyamines and polycarboxylates, even if the contribution of single donor groups to the stability of a given species is more difficult to quantify. 

As often mentioned, this kind of relationships may be exploited, for example, for a rough but fast estimation of the sequestration of dimethyltin(IV) cation by the organic matter (including humic and fulvic acids), just from the knowledge of parameters commonly measured during its characterization, such as the number of carboxylic and aminogroups. Of course, accuracy of estimated data is not as high as that of experimentally determined values, but their determination is certainly faster and simpler and gives an immediate first picture of the interactions occurring in the system of interest.

### 3.9. Literature Comparisons

Compared to the significant number of literature contributions on the biological activity/toxicity, the industrial and technological applications, and the environmental distribution of organotin(IV) compounds, relatively few papers (some are by this group) were published on their speciation, sequestration, and solution behavior in aqueous systems, where these compounds are most active. Among them, few report thermodynamic data (stability constants, formation enthalpies, and entropies, etc.) on interactions of alkyltin(IV) cations with aminoacids, and fewer with amines [7, 16, 22–32]. For these reasons, the most of the results reported in this paper should be considered as novel, making literature comparisons quite difficult to do. Nevertheless, some interesting features may be observed. Looking at previous studies by this group on dimethyltin(IV) complexes with other ligands of biological and environmental interest, it emerges that ligands containing amino groups generally show an intermediate sequestering ability between those having thiolic and carboxylic groups, for example, at *t* = 25°C, *I* = 0.1 mol L^−1^ and pH = 6.5, pL_50_ for *pei* is 3.18; whilst it is pL_50_ = 2.63 and pL_50_ = 4.39 for tricarballylic acid [19] and L-cysteine [31], respectively (*t* = 25°C, *I* = 0 mol L^−1^ and pH = 6). Important exceptions are represented by phytic acid [20] (pL_50_ = 4.12 at *t* = 25°C, *I* = 0.1 mol L^−1^ and pH = 6.5), whose sequestering ability is well known [63], and by carboxylic ligands containing other O-donor groups, like citric acid [19] (pL_50_ = 3.60 at *t* = 25°C, *I* = 0 mol L^−1^ and pH = 6) where the presence of an extra hydroxo-group seems to play an important role in complexation. 

Concerning stability constants of dimethyltin complexes with polyamines investigated in the present paper, in our knowledge no literature data are available. On the contrary, some values may be found for *dmt* complexes with some aminoacids, whose literature till years 2001-2002 was accurately reviewed in [7, 26]. The *dmt*-*gly* system was investigated by Shoukry in NaNO_3aq_ at *I* = 0.1 mol L^−1^ and *t* = 25°C [24], and by Surdy et al. [25] in the same conditions of ionic strength and temperature, but in NaClO_4aq_. The first author proposes a speciation scheme including the formation of ML and ML_2_ species, with log *β* = 8.76 and 15.92, respectively, whilst Surdy et al. reported the formation of ML, MLH, and MLOH species with corresponding log *β* = 7.99, 11.03, and 2.40, respectively, in good accordance with our values. In the same paper, Shoukry also determined the stability constants of ML, MLH, and ML_2_ species formed by lysine, with log *β* = 14.04, 19.35, and 18.52, respectively. 

Finally, it is also interesting to make some comparisons of the binding ability of other dialkyltin(IV) cations toward some of the ligands investigated in this work. In fact, it is already known from literature that chemicophysical behavior of alkyltin(IV) compounds regularly varies with the nature and number of alkyl groups bound to the central Sn(IV) atom, though the former factor is less important than the latter. As concerns dialkyltin(IV) cations, it was already observed that *dmt* and *det* behave similarly toward hydrolysis and complex formation with, for example, carboxylic and amino acids (including *gly* and *lys*, investigated here) [16, 24, 64]. From this point of view, the comparison with data reported in a previous study [16] by this group *det* interactions with *en*, *gly,* and *mal* is particularly significant, since they were obtained in the same experimental conditions. In the case of glycinate, the formation of MLOH species was not observed for *det*, whilst it was determined in the *dmt*/*gly* system, even if in small percentage. At the same time, *det*/*gly* species are more stable than the corresponding complexes formed by dimethyltin(IV) cation: at *I* = 0.1 mol L^−1^, for *dmt* we have log *K*_110_ = 7.74 and log *K*_111_ = 1.90, in the case of *det* it is log *K*_110_ = 9.07 and log *K*_111_ = 3.16. Regarding ethylenediamine these differences are less marked, so that log *K*_110_ and log *K*_111_ are slightly higher for *dmt* than for *det* (for *det*/*en* system we have log *K*_110_ = 10.38, log *K*_111_ = 5.79 and log *K*_120_ = 5.70).

## 4. Final Remarks

In the present paper, the sequestering ability of various polyamines and aminoacids of biological and environmental relevance toward dimethyltin(IV) cation was evaluated. The main conclusions can be summarized as follows:

- dimethyltin(IV) cation forms quite stable complexes with low and high molecular weight ligands containing amino- and/or carboxylic groups;
- in the experimental conditions used, all investigated amines form the ML and MLH species, whilst further ML_*q*_H_*r*_ with different values of *q *(*q* = 1 or 2) and *r *(*r* = 2 or 3) are formed, depending on the ligand;
- the three investigated aminoacids form the ML, MLH, and ML(OH) species; only *lys* also forms the diprotonated MLH_2_ species;
- the formation of these species ranges from ~10% to ~80%, indicating that they cannot be neglected in a correct study of dimethyltin(IV) speciation in real systems;
- the stability of complex species proved fairly dependent on ionic strength, and this dependence was modeled by a simple Debye-Hückel type equation and by the SIT approach;
- the sequestering ability of investigated ligands toward dimethyltin(IV) cation was defined by the calculation of several values of pL_50_, an empirical parameter able to give an objective representation of this binding ability;
- the sequestering ability of investigated ligands toward dimethyltin(IV) cation follows the trend *pei* > *paam* > *sper* ≅ *ptr* > *lys* > *en* > *asp* > *gly*;
- equations were formulated to model the dependence of pL_50_ on different variables, such as ionic strength and pH, and other empirical predictive relationships were also found between the stability of the complexes and the kind and number of functional groups of the ligand(s) involved in the formation equilibria.

## Figures and Tables

**Table 1:** Dimethyltin(IV) hydrolysis constants and protonation constants of ligands used, in NaCl_aq_ at *I* = 0.1 mol L^−1^ and *t* = 25°C. log *β*_pqr_ refer to equilibrium reported in (1).

Ligand	log *β*_011_	log *β*_012_	log *β*_013_	log *β*_014_	Reference
*en*	9.94	17.04	—	—	[51]
*ptr*	10.58	19.90	—	—	[51]
*paam*	9.74	17.51	—	—	[52]
*sper*	10.73	20.67	29.44	37.28	[51]
*pei*	9.36	17.48	23.19	25.69	[53]
*gly*	9.62	11.98	—	—	[57]
*asp*	9.65	13.36	15.30	—	[55]
*lys*	10.65	19.75	21.79	—	[56]
*dmt*					
log *β*_10−1_	log *β*_10−2_	log *β*_10−3_	log *β*_20−2_	log *β*_20−3_	
–3.12	–8.45	–19.44	–5.26	–9.61	[54]

**Table 2:** Stability constantsof dimethyltin(IV)/amine species, in NaCl_aq_ at different ionic strengths (in mol L^−1^) and *t* = 25°C. log *K*_*pqr*_ refer to equilibria reported in (2)–(4); ± standard deviation.

*I*/mol L^−1^	log *K*_110_	log *K*_120_	log *K*_111_	log *K*_121_	log *K*_112_	log *K*_113_
	*en *					
0.102	10.75 ± 0.02	4.86 ± 0.01	6.25 ± 0.02	—	—	—
0.253	10.70 ± 0.01	4.84 ± 0.01	6.32 ± 0.01	—	—	—
0.494	10.61 ± 0.01	4.81 ± 0.01	6.33 ± 0.01’	—	—	—
0.720	10.53 ± 0.02	4.79 ± 0.02	6.29 ± 0.01	—	—	—
0.948	10.44 ± 0.01	4.76 ± 0.03	6.24 ± 0.02	—	—	—
	*ptr *					
0.105	14.24 ± 0.02	3.46 ± 0.01	8.79 ± 0.02	—	—	—
0.243	14.19 ± 0.01	3.45 ± 0.02	8.85 ± 0.02	—	—	—
0.490	14.12 ± 0.01	3.41 ± 0.02	8.86 ± 0.02	—	—	—
0.722	14.04 ± 0.01	3.39 ± 0.03	8.81 ± 0.02	—	—	—
0.968	13.96 ± 0.02	3.36 ± 0.04	8.74 ± 0.03	—	—	—
	*paam*					
0.102	11.93 ± 0.02	—	7.46 ± 0.02	7.54 ± 0.03	—	—
0.252	11.94 ± 0.02	—	7.51 ± 0.02	7.67 ± 0.02	—	—
0.481	11.96 ± 0.01	—	7.47 ± 0.01	7.76 ± 0.02	—	—
0.725	11.97 ± 0.01	—	7.39 ± 0.01	7.81 ± 0.02	—	—
0.954	11.98 ± 0.01	—	7.29 ± 0.02	7.83 ± 0.03	—	—
	*sper *					
0.110	14.66 ± 0.02	—	12.80 ± 0.01	—	10.95 ± 0.03	7.06 ± 0.04
0.245	14.63 ± 0.01	—	12.88 ± 0.01	—	11.13 ± 0.02	7.35 ± 0.03
0.486	14.58 ± 0.01	—	12.91 ± 0.01	—	11.23 ± 0.02	7.52 ± 0.03
0.712	14.52 ± 0.02	—	12.89 ± 0.01	—	11.23 ± 0.02	7.55 ± 0.03
0.947	14.47 ± 0.01	—	12.86 ± 0.02	—	11.20 ± 0.03	7.53 ± 0.05
	*pei*					
0.101	11.92 ± 0.02	—	9.22 ± 0.02	—	5.35 ± 0.05	3.12 ± 0.08
0.249	11.88 ± 0.01	—	9.30 ± 0.01	—	5.51 ± 0.04	3.45 ± 0.06
0.501	11.81 ± 0.01	—	9.32 ± 0.01	—	5.53 ± 0.03	3.64 ± 0.04
0.752	11.75 ± 0.01	—	9.29 ± 0.01	—	5.46 ± 0.03	3.68 ± 0.05
0.999	11.68 ± 0.02	—	9.24 ± 0.02	—	5.36 ± 0.05	3.67 ± 0.08

**Table 3:** Stability constants of dimethyltin(IV)/aminoacid species, in NaCl_aq_ at different ionic strengths (in mol L^−1^), and *t* = 25°C. log *K*_*pqr*_ refer to equilibria reported in (2)–(4); ± standard deviation.

*I*/mol L^−1^	log *K*_110_	log *K*_111_	log *K*_112_	log *K*_11−1_
	*gly *			
0.100	7.74 ± 0.04	1.90 ± 0.04	—	5.60 ± 0.08
0.486	7.49 ± 0.03	1.34 ± 0.03	—	5.52 ± 0.06
	*lys*			
0.098	13.74 ± 0.07	9.01 ± 0.04	3.61 ± 0.06	6.66 ± 0.07
0.475	13.14 ± 0.05	7.97 ± 0.02	2.76 ± 0.09	7.10 ± 0.07
	*asp *			
0.098	8.00 ± 0.07	2.48 ± 0.03	—	5.84 ± 0.08
0.237	7.88 ± 0.06	2.43 ± 0.02	—	5.84 ± 0.07
0.482	7.94 ± 0.04	2.47 ± 0.02	—	5.96 ± 0.05
0.713	8.08 ± 0.04	2.56 ± 0.01	—	6.13 ± 0.05
0.958	8.28 ± 0.06	2.67 ± 0.02	—	6.32 ± 0.08

**Table 4:** Empirical parameters of (5) for the dependence of stability constants of dimethyltin(IV)/amine species on ionic strength in the molar or molal concentration scales, in NaCl_aq_ and *t* = 25°C.

*pqr*	log *K*_*pqr*_^(a,b)^	*C* ^(**b**)^	∆ε^(**b**)^
	*en*		
110	10.76 ± 0.01	−0.368 ± 0.009	−0.371 ± 0.012
111	6.25 ± 0.02	−0.442 ± 0.011	−0.451 ± 0.016
120	4.86 ± 0.03	−0.116 ± 0.012	−0.119 ± 0.010
	*ptr*		
110	14.24 ± 0.02	−0.316 ± 0.009	−0.328 ± 0.009
111	8.78 ± 0.03	−0.476 ± 0.015	−0.474 ± 0.011
120	3.46 ± 0.03	−0.117 ± 0.014	−0.121 ± 0.018
	*paam*		
110	11.93 ± 0.02	0.058 ± 0.013	0.044 ± 0.004
111	7.46 ± 0.03	−0.623 ± 0.015	−0.636 ± 0.016
121	7.54 ± 0.05	−0.087 ± 0.015	−0.106 ± 0.009
	*sper *		
110	14.67 ± 0.02	−0.235 ± 0.013	−0.236 ± 0.009
111	12.79 ± 0.01	−0.360 ± 0.018	−0.364 ± 0.0018
112	10.93 ± 0.02	−0.557 ± 0.017	−0.559 ± 0.015
113	7.02 ± 0.04	−0.716 ± 0.027	−0.722 ± 0.027
	*pei *		
110	11.92 ± 0.02	−0.284 ± 0.015	−0.272 ± 0.015
111	9.22 ± 0.01	−0.410 ± 0.021	−0.403 ± 0.018
112	5.34 ± 0.05	−0.850 ± 0.019	−0.834 ± 0.018
113	3.11 ± 0.07	−0.653 ± 0.026	−0.658 ± 0.024

^(a)^log *K*_*pqr*_ values at *I* = 0.1 (in both molar or molal concentration scales), taken as reference ionic strength; ^(b)^ ± standard deviation.

**Table 5:** Empirical parameters of (5) for the dependence of stability constants of dimethyltin(IV)/aminoacid species on ionic strength in the molar or molal concentration scales, in NaCl_aq_ and *t* = 25°C.

*pqr*	log *K*_*pqr*_^(a,b)^	*C* ^(*b*)^	∆ε^(*b*)^
	*gly *		
110	7.74	0.020	−0.001
111	1.90	−1.451	−1.454
11–1	5.60	0.126	0.102
	*lys *		
110	13.74	−0.911	−0.911
111	9.00	−2.759	−2.742
112	3.61	−2.936	−2.936
11–1	6.66	1.508	1.461
	*asp*		
110	7.99 ± 0.07	1.194 ± 0.015	1.165 ± 0.009
111	2.48 ± 0.03	0.653 ± 0.011	0.634 ± 0.012
11–1	5.84 ± 0.08	0.987 ± 0.021	0.963 ± 0.018

^(a)^log *K*_*pqr*_ values at * I* = 0.1 (in both molar or molal concentration scales), taken as reference ionic strength; ^(b)^ ± standard deviation; parameters for *gly* and *lys* species without errors, due to fits based on two experimental points.

**Table 6:** Stability constantsof dimethyltin(IV)/amine species, in NaCl_aq_ at different ionic strengths (in mol kg^−1^H_2_O) and *t* = 25°C. log *K*_*pqr*_ refer to equilibria reported in (2)–(4).

*I*/mol kg^−1^	log *K*_110_	log *K*_120_	log *K*_111_	log *K*_121_	log *K*_112_	log *K*_113_
	*en *					
0.102	10.75	4.86	6.25	—	—	—
0.255	10.70	4.83	6.32	—	—	—
0.500	10.60	4.81	6.32	—	—	—
0.732	10.52	4.79	6.28	—	—	—
0.968	10.43	4.75	6.23	—	—	—
	*ptr*					
0.106	14.24	3.46	8.79	—	—	—
0.245	14.19	3.44	8.85	—	—	—
0.496	14.11	3.41	8.85	—	—	—
0.734	14.03	3.39	8.80	—	—	—
0.988	13.95	3.35	8.74	—	—	—
	*paam*					
0.100	11.93	—	7.46	7.54	—	—
0.254	11.94	—	7.51	7.67	—	—
0.487	11.95	—	7.46	7.75	—	—
0.737	11.96	—	7.38	7.80	—	—
0.974	11.97	—	7.28	7.82	—	—
	*sper*					
0.111	14.66	—	12.80	—	10.95	7.06
0.247	14.63	—	12.88	—	11.13	7.35
0.500	14.57	—	12.90	—	11.22	7.51
0.732	14.51	—	12.88	—	11.22	7.54
0.968	14.46	—	12.85	—	11.19	7.52
	*pei*					
0.102	11.92	—	9.22	—	5.35	3.12
0.252	11.88	—	9.30	—	5.51	3.45
0.506	11.80	—	9.31	—	5.52	3.63
0.763	11.74	—	9.28	—	5.45	3.67
1.021	11.67	—	9.23	—	5.35	3.66

**Table 7:** Stability constantsof dimethyltin(IV)/aminoacid species, in NaCl_aq_ at different ionic strengths (in mol kg^−1^H_2_O), and *t* = 25°C. log *K*_pqr_ refer to equilibria reported in (2)–(4).

*I*/mol kg^−1^	log *K*_110_	log *K*_111_	log *K*_112_	log *K*_11−1_
	*gly*			
0.100	7.74	1.90	—	5.60
0.492	7.48	1.33	—	5.51
	*lys*			
0.098	13.74	9.01	3.61	6.66
0.481	13.14	7.96	2.75	7.09
	*asp*			
0.098	8.00	2.48	—	5.84
0.239	7.88	2.43	—	5.84
0.488	7.93	2.46	—	5.95
0.725	8.07	2.55	—	6.12
0.978	8.27	2.66	—	6.31

**Table 8:** Interaction coefficients of Specific ion Interaction Theory (SIT) equations for dmt and ligands species, at *t* = 25°C. ± standard deviation.

Cation	Anion	*ε*					
M^2+^	Cl^−^	−0.45 ± 0.01					
M(OH)^+^	Cl^−^	−0.106 ± 0.008					
M(OH)_2_	—	0.018 ± 0.009					
		*en*	*ptr*	*paam*	*sper*	*pei*	*asp*

L	—	0	0	0	0	0	
LH^+^	Cl^−^	−0.154 ± 0.004	−0.088 ± 0.006	−0.56 ± 0.03	−0.190 ± 0.008	−0.43 ± 0.05	
LH_2_^2+^	Cl^−^	−0.122 ± 0.004	−0.223 ± 0.007	−1.09 ± 0.05	−0.31 ± 0.02	−0.88 ± 0.06	
LH_3_^3+^	Cl^−^	—	—	—	−0.27 ± 0.03	−1.09 ± 0.07	
LH_4_^4+^	Cl^−^	—	—	—	−0.20 ± 0.05	−0.62 ± 0.098	
ML^2+^	Cl^−^	−0.079 ± 0.003	−0.122 ± 0.004	−0.494 ± 0.006	−0.205 ± 0.006	−0.18 ± 0.01	
MLH^3+^	Cl^−^	−0.153 ± 0.007	−0.056 ± 0.008	−0.37 ± 0.03	−0.25 ± 0.05	−0.48 ± 0.05	
MLH_2_^4+^	Cl^−^	—	—	—	−0.18 ± 0.07	−0.50 ± 0.07	
MLH_3_^5+^	Cl^−^	—	—	—	0.02 ± 0.09	0.88 ± 0.07	
ML_2_^2+^	Cl^−^	0.042 ± 0.006	0.001 ± 0.006	—			
ML_2_H^3+^	Cl^−^	—	—	−1.46 ± 0.06			
Na^+^	L^2−^						0.20 ± 0.02
Na^+^	LH^−^						0.025 + 0.009
LH_2_	—						0.012
ML	—						−1.40 ± 0.02
MLH^+^	Cl^−^						−1.06 ± 0.01
Na^+^	MLOH^−^						−0.87 ± 0.02

**Table 9:** pL_50_ values for the sequestration of *dmt* by various ligands, at *I* = 0.1 mol L^−1^, *t* = 25°C and different pH.

pH	pL_50_^(a)^							
	*en*	*ptr*	*paam*	*sper*	*pei*	*gly*	*asp*	*lys*
4.5	2.24	2.60	2.80	2.42	3.11	1.30	1.53	3.03
5.5	2.20	2.72	2.85	2.66	3.46	1.29	1.50	2.78
6.5	2.10	2.78	2.89	2.76	3.18	1.31	1.52	2.51
7.0	2.05	2.78	2.91	2.76	2.99	1.31	1.52	2.46
8.1	2.07	2.76	2.77	2.62	2.60	1.31	1.51	2.40

^(a)^ ± 0.01-0.02 standard deviation.

**Table 10:** pL_50_ values for the sequestration of *dmt* by various ligands, at pH = 6.5, *t* = 25°C and different ionic strengths.

*I*/mol L^−1^	pL_50_^(a)^					
	*en*	*ptr*	*paam*	*sper*	*pei*	*asp*
0.10	2.10	2.78	2.89	2.76	3.18	1.52
0.25	2.10	2.76	2.67	2.70	3.06	1.61
0.50	2.10	2.75	2.48	2.66	2.96	1.82
0.75	2.10	2.72	2.37	2.69	2.97	2.05
1.00	2.09	2.70	2.32	2.77	2.87	2.32

^(a)^ ± 0.01-0.02 standard deviation.

**Table 11:** Dataset of protonation, complex formation constants and number of functional groups involved in the complex formation reaction for *dmt*/amine and *dmt*/carboxylate species, at *t* = 25°C and infinite dilution, used to derive parameters of (16) and (17).

*pqr*	ligand	log *K*_*pqr*_	log *K*_01(*r*+1)_	n_N_	n_O_
110	*en*	10.79	9.90	2	0
111	*en*	5.85	6.87	1	0
110	*ptr*	14.27	10.54	2	0
111	*ptr*	8.39	9.10	1	0
110	*sper*	14.69	10.77	4	0
111	*sper*	12.39	9.69	3	0
112	*sper*	10.10	8.38	2	0
113	*sper*	5.78	7.28	1	0
110	*pei*	11.95	9.36	4	0
111	*pei*	8.82	7.90	3	0
112	*pei*	4.55	5.29	2	0
113	*pei*	1.86	1.80	1	0
110	*paam*	11.93	9.69	2	0
111	*paam*	7.08	7.80	1	0
110	*ac*	3.01	4.74	0	1
110	*mal*	5.43	5.70	0	2
111	*mal*	2.11	2.86	0	1
110	*Succ*	4.98	5.64	0	2
111	*succ*	2.94	4.21	0	1
110	*tca*	6.69	6.49	0	3
111	*tca*	4.63	4.91	0	2
112	*tca*	2.98	3.68	0	1
110	*btc*	8.20	7.18	0	4
111	*btc*	6.16	5.83	0	3
112	*btc*	4.46	4.53	0	2
113	*btc*	2.86	3.38	0	1
